# Aerobic Fitness Linked to Cortical Brain Development in Adolescent Males: Preliminary Findings Suggest a Possible Role of BDNF Genotype

**DOI:** 10.3389/fnhum.2016.00327

**Published:** 2016-06-30

**Authors:** Megan M. Herting, Madison F. Keenan, Bonnie J. Nagel

**Affiliations:** ^1^Department of Pediatrics, Children’s Hospital Los AngelesLos Angeles, CA, USA; ^2^Department of Behavioral Neuroscience, Oregon Health & Science UniversityPortland, OR, USA

**Keywords:** exercise, BDNF, genotype, neuroimaging, adolescence, cortical volume

## Abstract

Aerobic exercise has been shown to impact brain structure and cognition in children and adults. Exercise-induced activation of a growth protein known as brain derived neurotrophic factor (BDNF) is thought to contribute to such relationships. To date, however, no study has examined how aerobic fitness relates to cortical brain structure during development and if BDNF genotype moderates these relationships. Using structural magnetic resonance imaging (MRI) and FreeSurfer, the current study examined how aerobic fitness relates to volume, thickness, and surface area in 34 male adolescents, 15 to 18 years old. Moreover, we examined if the val66met BDNF genotype moderated these relationships. We hypothesized that aerobic fitness would relate to greater thickness and volumes in frontal, parietal, and motor regions, and that these relationships would be less robust in individuals carrying a Met allele, since this genotype leads to lower BDNF expression. We found that aerobic fitness positively related to right rostral middle frontal cortical volume in all adolescents. However, results also showed BDNF genotype moderated the relationship between aerobic fitness and bilateral medial precuneus surface area, with a positive relationship seen in individuals with the Val/Val allele, but no relationship detected in those adolescents carrying a Met allele. Lastly, using self-reported levels of aerobic activity, we found that higher-fit adolescents showed larger right medial pericalcarine, right cuneus and left precuneus surface areas as compared to their low-fit peers. Our findings suggest that aerobic fitness is linked to cortical brain development in male adolescents, and that more research is warranted to determine how an individual’s genes may influence these relationships.

## Introduction

Adolescence is a time period of significant neurodevelopment. Using structural magnetic resonance imaging (MRI), cortical volume has been shown to follow an inverted U-shaped pattern over development (Raznahan et al., 2011), with increases seen across childhood, peaking around early adolescence, and then decreasing before stablizing in adulthood (Giedd et al., 1999; Giedd, 2004). Within this measurement of cortical volume, two different aspects of the cortical sheet are also reflected: cortical thickness and surface area (Dale et al., 1999; Winkler et al., 2010, 2012). Cortical thickness is a measurement of the cortical ribbon, as defined as the distance between white matter and pial surfaces at each voxel; whereas surface area is defined as the area of the exposed cortical pial surface and hidden area of cortex within the sulci (Dale, 1999; Fischl et al., 2002, 2004). While related, cortical volume, thickness, and surface area are unique metrics showing distinct cellular, biological, and evolutionary relationships (Raznahan et al., 2011). The dynamic changes in cortical morphology across adolescence highlight the extraordinary plasticity of the brain during this period of development.

Given that the brain is still undergoing remodeling into the third decade of life, the developing adolescent brain is thought to be especially sensitive to environmental factors (Masten, 2004; Marco et al., 2011). In this regard, aerobic exercise is one environmental factor that may influence the developing adolescent brain, as it has been previously linked to brain and behavior improvements. Both cross-sectional and intervention studies have found aerobic exercise and fitness to relate to better performance on cognitive tasks in children, adolescents, and adults. For example, aerobic fitness has been associated with better learning and memory in children (Chaddock et al., 2010a, 2011), adolescents (Herting and Nagel, 2012), and the elderly (Erickson et al., 2011). Moreover, higher-fit children show better performance on tasks of executive functions, such as attention, compared to low-fit children (Chaddock et al., 2011, 2012a; Voss et al., 2011). Similar results have also been reported in older adults (Erickson and Kramer, 2009; Voss et al., 2012). These behavioral findings are mirrored by associations between aerobic fitness and brain volume. For example, better memory parallels positive correlations between aerobic fitness and larger hippocampus sizes across the lifespan (children (Chaddock et al., 2010a); adolescents (Herting and Nagel, 2012); and older adults (Erickson et al., 2011)). Furthermore, larger caudate volumes in children (Chaddock et al., 2010b) and older adults (Verstynen et al., 2012) have been shown to relate to aerobic exercise-related performance in executive functioning. However, relatively less research has examined how aerobic fitness relates to cortical gray matter brain volume across the lifespan. Using voxel-based morphometry, a 6-month aerobic exercise intervention study in older adults (ages 60–79 years) found increases in gray matter tissue density following intervention in the frontal, parietal, and temporal lobes compared to a non-aerobic control group (Colcombe et al., 2006). However, the same study showed that aerobic exercise did not predict increases in gray matter in a young adult sample (ages 18–30 years; Colcombe et al., 2006). Furthermore, despite differences in subcortical volumes, high and low-fit children were found to have similar total gray matter volumes (Chaddock et al., 2010b). To our knowledge, no study to date has examined how aerobic fitness relates to region specific differences in cortical gray matter volume, cortical thickness, or cortical surface area. Moreover, the current discrepancies in the brain-exercise relationships across different samples could be due to differences in the limited quantification variables used to assess cortical structure (total gray matter volume, voxel-based morphometry). Thus, a more thorough assessment of how aerobic fitness relates to gray matter volume is warranted. Specifically, given the dynamic cortical changes that occur across adolescence, the unique and important relationship between aerobic fitness and cortical structure during this time deserves further investigation.

In addition to examining the influence of environmental factors (i.e., aerobic exercise), it is widely believed that complex genetic and environmental interactions exist to determine an individual’s cortical neurodevelopment. Brain-derived neurotrophic factor (BDNF), which plays a role in synaptic plasticity and cell growth and survival throughout the cortex, is thought to be vital in terms of exercise’s influence on brain structure (for review see Cotman et al., 2007; van Praag, 2008, 2009). A three to four-fold increase is seen in BDNF following exercise (Neeper et al., 1995; Cotman and Berchtold, 2002), which continues after several weeks of exercise (Russo-Neustadt et al., 1999). BDNF has also been shown to mediate the effect of exercise on brain and cognition in animals (Vaynman et al., 2004; Llorens-Martin et al., 2008). Individual differences in the BDNF gene, however, may also influence how exercise affects brain and behavior. The secretion and intracellular trafficking of BDNF is altered by a common functional single nucleotide polymorphism (SNP) within the BDNF gene, known as the val66met (Egan et al., 2003). Specifically, an amino acid substitution of the Valine (Val) to Methionine (Met) can occur at codon 66 of the prodomain of the BDNF gene. The Met substitution has been shown to lead to decreases in activity-dependent secretion of BDNF as compared to the Val allele (Egan et al., 2003). The val66met genotype has been associated with regional cortical surface, thickness, and volume in adults (Pezawas et al., 2004; Wang et al., 2014); although, negative findings have been noted (Jessen et al., 2009; Koolschijn et al., 2010). One study recently showed that the benefits of exercise on cognition are moderated by BDNF allele status. Specifically, a four week exercise intervention resulted in improvements in cognition on an object recognition memory task in Val/Val carriers, but not in those individuals with the Met substitution (Hopkins et al., 2012). Therefore, it is possible that the influence of aerobic fitness on cortical development may be further influenced by an individual’s BDNF genotype.

The goals of the current study were to examine the association between aerobic fitness and cortical gray matter morphometrics in adolescents, including gray matter volume, surface area, and thickness. Given previous research showing aerobic exercise leads to better cognition and greater gray matter density in regions subserving cognitive function (Colcombe et al., 2006), we hypothesized that aerobic fitness would relate to greater thickness and volumes in frontal and parietal regions. Furthermore, we hypothesized that the relationships between aerobic fitness and cortical structure would be reduced in individuals with the Met substitution (Val/Met and Met/Met) as compared to those with a Val/Val BDNF genotype.

## Materials and Methods

### Participants

Thirty-four eligible male youths, ages 15–18, participated in the study as part of a more comprehensive neuroimaging study on exercise and the adolescent brain, including diffusion tensor imaging (Herting et al., 2014), hippocampal structure and function (Herting and Nagel, 2012) and functional magnetic resonance imaging (fMRI) verbal memory encoding (Herting and Nagel, 2013). All procedures of the current project were approved from Oregon Health & Science University’s Institutional Review Board and conducted in accordance with the Declaration of Helsinki. The study was adversitised through fliers, advertisements, and mailers circulated throughout the community. Participants and one of their parents underwent comprehensive structured interviews via telephone to determine eligibility after obtaining written consent and assent from all youths and at least one biological parent.

As previously reported, participants also had to meet either high or low-fit criteria via self-report on a modified version of the Youth Adolescent Activity Questionnaire (Wolf et al., 1994; Rifas-Shiman et al., 2001; for full details see Herting and Nagel (2013). Briefly, high-fit youth were defined as those participating in an average of ≥10 h per week of regular, organized aerobic physical activity, purposely performed to allow for improvement or maintenance of aerobic fitness across one or more seasons, within the past year. Low-fit youth were defined as those individuals that had participated in ≤1.5 h of aerobic physical activity per week over the past year. High-fit youth were asked to participate in the study during the season in which they were most physically active based on their YAAQ self-report. These criteria were set forth, as significant increases in aerobic fitness have been seen in adolescents who participated in ≥10 h of aerobic exercise per week (Brown et al., 1972; Weber et al., 1976; Lussier and Buskirk, 1977), and relatively extreme categorizations (≥10 vs. ≤1.5 h per week) maximize the likelihood of detecting group differences.

Particpants were excluded if they had significant substance abuse (>10 lifetime alcoholic drinks or 2 drinks/occasion, >5 uses of marijuana, any other drug use, or >4 cigarettes per day) [Brief Lifetime version of the Customary Drinking and Drug Use Record (Brown et al., 1998)]; a currently diagnosed DSM-IV psychiatric disorder [Diagnostic Interview Schedule for Children Predictive Scales (DISC-PS-4.32b; Lucas et al., 2001; Hoven et al., 2005)]; reported history of psychotic disorders in biological parents [Family History Assessment Module (FHAM; Rice et al., 1995)]; major medical condition or significant head trauma [Structured Clinical Interview (SCI; Brown et al., 1994)]; left-handedness [Edinburgh Handedness Inventory (Oldfield, 1971)], or irremovable metal. The current study was limited to recruitment of male adolescent participants. There are many inherent differences due to sex in brain structure [thickness, surface area, and volume] (Sowell et al., 2007; Lenroot and Giedd, 2010; Bramen et al., 2011, 2012) and aerobic capacity and physical fitness (Ekelund et al., 2001). As a consequence of these sex-specific disparaties, we chose to address the relationship of aerobic capacity and gray matter thickness, surface area, and volume in males only, thereby reducing variability within the study population for this preliminary work.

#### Demographic Information and Potential Theoretical Confounds

During the structured telephone interviews, information on age, ethnicity, and grade point average (GPA) was gathered. In order to account for non-exercise related factors that are also shown to influence cortical maturation, we assessed additional lifestyle factors. The Hollingshead Index of Social Position (ISP) was administered to parents to asses socioeconomic status (SES) based on each parents’ occupation and educational attainment (Hollingshead, 1975). Each participant also provided self-report of their pubertal status [Pubertal Development Scale (PDS; Petersen et al., 1988)], and personal lifestyle habits involving nutrition, safety, relaxation, health promotion, and substance use [Revised Personal Lifestyle Questionnaire (PLQ; Mahon et al., 2003)]. These factors were assessed as SES (Noble et al., 2015), pubertal hormones (Herting et al., 2015), and a number of lifestyle factors (e.g., substance use) have been shown to relate to cortical volumes during adolescence (Jacobus et al., 2014, 2015; Squeglia et al., 2014, 2015).

### Procedure

Over a 15-day time window, youths completed: (1) an aerobic fitness and physical activity assessment; (2) a blood-draw to determine BDNF genotyping; and (3) a MRI scanning session at Oregon Health & Science University (OHSU).

#### Aerobic Capacity Testing

A computerized indirect calorimetry system (VMax Series, V6200 Autobox, Sensormedics, VIASYS Healthcare) assessed aerobic fitness of each participant by determining peak oxygen uptake rates (VO_2_ peaks) during exercise on a Bruce Protocol (Bruce et al., 1973). VO_2_ peak is considered the most valid objective measurement of aerobic physical fitness. The peak signifies the highest rate of oxygen transportation and utilization in the body during incremental exersize (Armstrong and Welsman, 2007). Participants ran on a motor-driven treadmill starting at 1.7 mph with a 10% incline, with the speed and incline increasing every 3 min, while participant heart rate was continuously measured. Additionally, every 2 min, self-perceived measures of exertion were assessed on a scale of 0 (very easy) to 10 (very hard) until the particant reported exhaustion. Validity of VO_2_ peaks was required for consideration and determined by meeting at least one of the physiological criteria determining maximal effort outlined by Armstrong and van Mechelen (2008): (1) a plateau in oxygen consumption signifying that oxygen consumption remained at a steady state despite an increase in workload; (2) heart rate reached ≥ 200 beats/min; (3) the respiratory exchange ratio ≥ 1.0; and/or the subjective criteria of reporting a 10 on the perceived exertion scale. Just before aerobic testing participants’ lean body mass (LBM) was determined using a biolectrical impedence test with a Body Composition Analyzer, Model 310e (Biodynamics Corp, Seattle, WA, USA). The independent measure of aerobic fitness was expressed as peak oxygen consumption in mL/kg LBM/min, as it allows for body fat to be minimized as a confounding variable in expressing fitness as it relates to metabolism in youths (Dencker et al., 2010).

#### BDNF Genotyping

Blood was obtained via venapuncture by a trained phlebotomist at Oregon Clinical and Translational Research Center (OCTRI) at OHSU. Specimens were stored and genotyping was performed by the OCTRI core laboratory. Specifically, genomic DNA was extracted from whole blood using the Puregene system (Qiagen, Valencia, California, USA). The Val66Met SNP in the BDNF gene was genotyped by polymerase chain reaction (PCR) using primer sequences 5′-CAAACATCCGAGGACAAGGT-3′ and 5′-CCTCATGGACATGTTTGCAG-3′ and ABI Amplitaq Gold 360 PCR kits (Applied Biosystems, Foster City, California, CA, USA). Following PCR the derived DNA product samples were sequenced using ABI BigDye v3.1 DNA sequencing kits following the manufacturer’s procedures and analyzed on an ABI PRISM Genetic Analyzer 3130 × L.

The frequency of the BDNF val66met allele in this male and predominantly caucasian group of adolescents was was 62.5% Val/Val, 31% Met/Val, and 6.3% Met/Met, which is similar to frequencies observed in a large sample of Caucasian men (Pivac et al., 2009). The frequency of the val66met allele also did not signifcantly differ between high and low-fit participants (*X*^2^ = 2.2, *p* = 0.33). However, given the few participants homozygous for the Met allele and previous findings that at least one Met-allele has been linked to brain structure (Pezawas et al., 2004) and cognition (Hopkins et al., 2012), participants in the current study with at least one Met allele were combined into a single “Met allele carriers” group.

### MRI Scanning Session

All images were acquired at OHSU’s Advanced Imaging Research Center on a 3.0 Tesla Siemens Magnetom Tim Trio system (Siemens Medical Solutions, Erlangen, Germany) with a 12-channel head coil. Whole-brain T1 weighted MPRAGE scanning sequence was collected in the sagittal plane (Inversion Time (TI) = 900 ms, Flip Angle = 10 degrees, Echo Time (TE) = 3.58 ms, Repetition Time (TR) = 2300 ms, acquisition matrix = 256 × 240, 160 slices, resolution = 1 × 1 × 1.1 mm).

#### Image Processing and Statistical Analyses

Images were analyzed with Freesurfer v5.3, which has been validated against histological analysis (Rosas et al., 2002). Images were processed for skull stripping (Segonne et al., 2004), automated registration to Talairach space, segmentation of the subcortical regions (Fischl et al., 2002, 2004), segmentation of gray, white, and CSF tissue, and intensity normalization (Dale and Sereno, 1993; Dale et al., 1999; Fischl and Dale, 2000). The surfaces were inflated (Fischl et al., 1999a) and registered to a spherical atlas which matched cortical geometry across subjects by utilizing individual cortical folding patterns (Fischl et al., 1999b), the cerebral cortex was portioned into gyri and sulci (Fischl et al., 2004; Desikan et al., 2006), and a cortical surface map was created based on curvature and sulcal depth. Image analysis was based on an intensity and continuity distribution from the entire MRI volume in segmentation and deformation procedures to produce representations of cortical thickness (Fischl and Dale, 2000). During map creation spatial intensity gradients were also used for each tissue class rather than absolute signal intensity. Lastly, each participant’s brain was registered onto the Freesurfer average space and smoothed with a 10 mm full width at half maximum (FWHM) kernel. Each step of preprocessing was checked manually by MFK who was blind to participant demographics (aerobic fitness condition, age, PDS). Intracranial volume (ICV) was extracted and examined between the groups and as a function of VO_2_ peak as a potential covariate for volume analyses.

All statistical analyses of demographic variables were carried out using R Software (R Development Core Team, 2008). Normality was verified on all variables, and transformations were used when appropriate. When data continued to violate normality, nonparametric tests were employed. Students *t*-tests and Mann Whitney *U* tests were used to examine between group differences on demographic variables. Pearson correlations were also used to determine if these potential confounding variables related to aerobic fitness (VO_2_ peak). After identifying covariate(s) using R, FreeSurfer’s general linear model QDEC program (QDEC^1^) was used to perform whole brain vertex-based analyses for the dependent variables of interest, including average cortical thickness, surface area, and volume. For volume, thickness and surface area, the influence of aerobic fitness (i.e., VO_2_ peak) was examined, while controlling for potential confounding variables. Given that ICV was neither significantly different between HF and LF groups nor related to VO_2_ peak (*p*′s > 0.64), it was not included in the model for volume. To correct for multiple comparisons, Monte Carlo corrections were applied, and significance was determined at those regions with *p-values* < 0.05 after correction. Only results that passed multiple corrections are reported below.

## Results

### Participant Characteristics

Participant characteristics can be found in Table 1. One participant’s parent (low-fit) chose not to disclose total household income, one subject (high-fit) did not complete the PLQ, and two subjects (one low-fit, one high-fit) did not consent for storing blood to assess DNA; resulting in pairwise missing data for these measures. Significant group differences were seen in SES and PDS (Table 1). Pearson correlations were also used to determine if these potential confounding variables related to aerobic fitness (VO_2_ peak). No relationship was detected between SES and VO_2_ peak, but PDS was shown to be negatively correlated with VO_2_ peak (*r* = −0.37, *t*_(32)_ = 2.25, *p* = 0.03). Thus, PDS and SES were included as covariates during between-group statistical testing, while PDS was included as a covariate when examining relationships between brain and the continuous variable of aerobic fitness (VO_2_ peak).

**Table 1:** **Participant demographics**.

Demographics	All	HF	LF	HF vs. LF
N	34	17	17	
Age	15.9 (0.9)	16.6 (0.8)	16.2 (0.8)	*t*_(31.9)_ = 1.36, *p* = 0.18
% Caucasian	82.4	82.4	82.4	
IQ^a^	117.7 (9.6)	117.1 (11.8)	118.0 (7.1)	*t*_(26.1)_ = 0.26, *p* = 0.79
SES^b^	22.4 (10.8)	18.3 (6.0)	26.5 (12.9)*	*t*_(22.6)_ = 2.39, *p* = 0.03
Median household income^b^ (Thousands)	113	130	90^en^	
Puberty^c^	3.2 (0.4)	3.06 (0.4)	3.3 (0.3)*	*U* = 80, *z* = 2.24, *p* = 0.03
**BDNF**
Genotype*
Val/Val	20 (62.5%)	9	11	*X*^2^ = 2.2, *p* = 0.33
Met carriers	12 (37.5%)	7	5	
**Daytime activity levels and aerobic fitness**
Aerobic activity (h/wk over past year)^d^	5.5 (5.8)	11.3 (3.4)	0.26 (0.5)**	*t*_(16.7)_ = 12.61, *p* < 0.001
Aerobic activity (h/wk in season scanned)^d^	6.4 (6.8)	12.6 (3.8)	0.24 (0.5)**	*t*_(16.5)_ = 13.39, *p* < 0.001
VO_2_ peak (mL/kg LBM/min)	72.4 (10.5)	77.7 (10.5)	67.0 (7.4)*	*t*_(28.6)_ = 3.41, *p* = 0.002
**Body composition**
BMI^e^	22.0 (3.4)	21.3 (2.4)	22.7 (4.1)	*t*_(25.76)_ = 1.27, *p* = 0.21
**Lifestyle**
Nutrition^*f*¥^	12.2 (1.3)	12.0 (1.0)	12.4 (1.5)	*t*_(31)_ = 0.77, *p* = 0.45
Relaxation^*f*¥^	15.3 (2.2)	15.2 (2.2)	15.4 (2.2)	*t*_(31)_ = 0.22, *p* = 0.83
Health promotion^*f*¥^	13.3 (1.9)	13.7 (1.3)	13.0 (2.3)	*U* = 117, *z* = 0.70, *p* = 0.51
Safety^*f*¥^	14.6 (1.3)	14.2 (1.3)	15.0 (1.2)	*U* = 86.5, *z* = 1.8, *p* = 0.07
Substance use^*f*¥^	11.5 (0.8)	11.6 (0.5)	11.3 (1.0)	*U* = 122, *z* = 0.58, *p* = 0.63
**Extracurricular activities**
Frequency	3.8 (0.7)	4 (0)	3.5 (1.0)	*U* = 110.5, *z* = 2.09, *p* = 0.25
Number	2.62 (1.3)	2.9 (1.3)	2.3 (1.3)	*U* = 109, *z* = 1.28, *p* = 0.23

Means and standard deviations unless otherwise noted. ^¥^*n* = 16 due to missing data; *p < 0.05; **p < 0.01; h/wk = hours per week. ^a^Wechsler Abbreviated Scale of Intelligence. ^b^Hollingshead Index of Social Position; lower values reflect higher SES. ^c^Pubertal Development Scale. ^d^Youth Adolescent Activity Questionnaire. ^e^Body Mass Index. ^f^Personal Lifestyle Questionnaire. *N = 32.

### Cortical Structure

#### Group Differences

**Figure 1:**
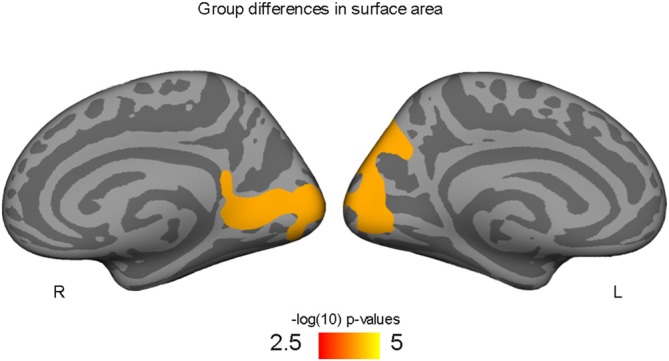
**Occipital and precuneus regions where high-fit youth have larger cortical surface areas compared to low-fit youth, controlling for both SES and PDS variables**.

Higher-fit adolescents showed larger right medial pericalcarine and cuneus and left precuneus surface areas as compared to low-fit adolescents (Table 2; Figure 1).

**Table 2:** **Cortical structures showing: (a) group differences (high or low fitness); or (b) a relationship with aerobic fitness (VO_2_ peak)**.

	Region	*X*	*Y*	*Z*	Size (mm^2^)	*T*	Cohen’s *d*
**(a) Group differences**
Surface area
Left	Precuneus	−12.0	−67.1	34.7	3551.89	4.00	1.51
Right	Pericalcarine	14.3	−77.4	4.8	3281.16	4.00	1.51
**(b) VO_2_ peak**
Volume
	Rostral
Left	Middle	−37.3	23.6	24.8	1083.62	2.14	0.77
	Frontal

**Figure 2:**
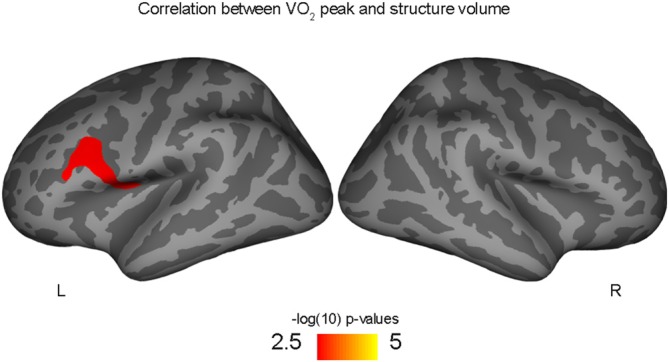
**Positive correlation between left rostral middle frontal volume and aerobic fitness (VO_2_ peak), controlling for PDS variable**.

**Figure 3:**
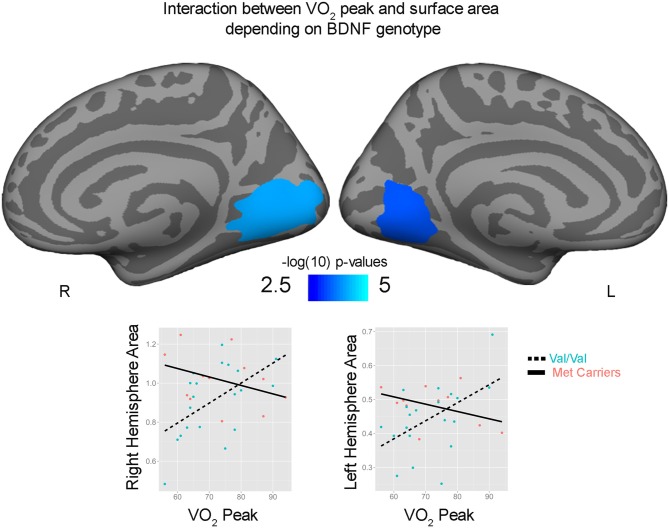
**Interaction between bilateral lingual gyrus surface area and VO_2_ peak depending on brain derived neurotrophic factor (BDNF) genotype.** Surface area (mm^2^) and VO_2_ peak are plotted for Val/Val (blue circle, dashed lines) and for Met allele carriers (pink, solid lines) from peak locations of the bilateral significant clusters.

Correlations with aerobic fitness: a significant positive relationship was seen between VO_2_ peak and left rostral middle frontal cortical volume (Table 2; Figure 2). A significant VO_2_ peak and BDNF genotype interaction was found in bilateral lingual gyrus surface area (Table 3; Figure 3). Specifically, a significant positive relationship was seen between VO_2_ peak and surface area in those with the Val/Val genotype (right hemisphere: *r* = 0.54, *t*_(18)_ = 2.71, *p* = 0.01; left hemisphere: *r* = 0.50, *t*_(18)_ = 2.48, *p* = 0.02), whereas this relationship was not significant between VO_2_ peak and lingual gyrus surface area in Met allele carriers (right hemisphere: *r* = −0.37, *t*_(10)_ = 1.23, *p* = 0.24; left hemisphere: *r* = −0.44, *t*_(10)_ = 1.57, *p* = 0.15).

**Table 3:** **Interaction between BDNF genotype and the correlation between structural surface area and VO_2_ peak**.

	Region	*X*	*Y*	*Z*	Size (mm^2^)	*T*	Cohen’s *d*
**VO_2_ peak**
Surface area
Left	Lingual gyrus	−21.4	−61.3	8.9	2300.80	−3.10	−1.22
Right	Lingual gyrus	14.3	−77.4	4.8	3496.36	−4.00	−1.57

## Discussion

These findings show for the first time that aerobic fitness relates to cortical surface area and volume in adolescent male youth. Congruent with our *a priori* hypothesis that aerobic fitness would relate to larger cortical morphometrics in frontal and parietal regions, we found that greater aerobic fitness (VO_2_ peak) predicted larger left middle prefrontal cortex volumes. In addition to our hypothesized prefrontal and parietal findings, we also found higher-fit adolescents had larger left precuneus and right occipital surface areas compared to their low-fit peers. Given that BDNF is thought to be responsible for exercise-related neuroplasticity, we also hypothesized that individual differences in the functional SNP within the BDNF gene may moderate how aerobic exercise related to cortical brain structure in this sample of male adolescents. Indeed, we found preliminary evidence that aerobic fitness predicted larger lingual gyrus surface area for those with the Val/Val BDNF genotype, but a positive relationship was not seen in Met allele carriers.

After a peak in volume and surface area during late childhood and early adolescence, cortical maturation includes a reduction in gray matter volume and a contraction (or decrease) of the surface area across the latter period of adolescence (Giedd et al., 1999; Giedd, 2004). The developmental trajectory, however, varies greatly by brain region, with sensory and motor regions peaking earlier as compared to prefrontal and parietal cortices that peak later in adolescence (Sowell et al., 2004). While the exact mechanisms underlying changes in cortical volume and surface area during adolescence remains to be determined, it is thought the inverted-U pattern reflects a proliferation of synapses followed by a pruning period (Lenroot and Giedd, 2006). Based on the known associations between aerobic fitness and executive function, we hypothesized that aerobic fitness may especially relate to larger cortical volumes, thickness, and surface area within the frontal and parietal lobes, since these regions are last to undergo maturation during this critical period of neuroplasticity. Along these lines, a positive association was found between aerobic fitness and frontal and parietal cortices in the current sample of adolescent males. These frontal and parietal findings are in agreement with previous studies. One study found that after completing a 6-month exercise intervention which required three 1-h exercise training sessions per week, elderly individuals (60–79 years) showed an increase in VO_2_ peak, as well as increases in lateral prefrontal and parietal cortical volumes (Colcombe et al., 2006). In addition, the anatomical locations of our findings are in line with the larger body of literature suggesting aerobic fitness may benefit cognition (Erickson and Kramer, 2009; Voss et al., 2011; Chaddock et al., 2012b). The rostral middle frontal cortex (also known as the dorsal lateral prefrontal cortex) and the precuneus are brain regions known to be important for executive functions, including attention and working memory (Wager and Smith, 2003; Funahashi, 2006; Parks and Madden, 2013). However, despite these similarities, it is interesting to note that other studies have found no significant differences in gray matter volumes between high vs. low-fit pre-pubertal children (Chaddock et al., 2010b). Similarly, no changes in gray matter volumes were found in young adults (18–30 years) following the aforementioned 6-month exercise intervention program that lead to volumetric changes in the elderly sample (Colcombe et al., 2006). Although the research between aerobic fitness and cortical size is limited, a discrepancy in results across samples could be due to the use of different methods. For example, the current study examined cortical volume and surface area measured by Freesurfer, as compared to the elderly and adult studies, which used voxel based morphometry. In addition, the child study by Chaddock et al. (2010b) used Freesurfer to examine high and low-fit children, but results may have been confounded by the low-fit children having higher BMI (a variable on which our groups were not significantly different), as BMI and obesity have been shown to relate to brain volumes in other samples (Yokum et al., 2012; Alosco et al., 2014; Ou et al., 2015). Alternatively, it is also feasible that aerobic exercise may have varying effects on the brain as a function of age, with larger effects seen during dynamic remodeling or age-related decline, such as adolescence and aging, respectively.

Because sensorimotor regions are typically thought to mature earlier in adolescence, we did not expect to see group differences, as well as a significant fitness-by-genotype interaction, in brain regions important for visual processing, including the occipital cortex and lingual gyrus. However, in a more recent longitudinal MRI study, surface areas of occipital regions (including the precuneus and lingual gyrus) were found to peak between 11 and 20 years of age, suggesting these visual areas also remain dynamic as well across adolescence (Vijayakumar et al., 2016). Moreover, while the lingual gyrus is involved in vision, its role in brain function likely goes beyond basic sensory processing. Through its interactions with the prefrontal cortex, the lingual gyrus has been implicated in modulating selective visual attention (Hopfinger et al., 2000; Vuilleumier et al., 2005). Thus, vantage points, visual search, anticipation, and fast reaction times are visual properties that may be essential to sports and engaging in various types of aerobic exercise. One hypothesis could be that aerobic exercise is associated with occipital lobe structure due to both basic, as well as higher-order visual abilities required and/or affected by exercise participation. However, given the cross-sectional design of the current study, it remains to be determined if aerobic exercise leads to greater surface area and volumes in these regions across adolescence, or if cortical size in these regions may in fact predispose children to perform aerobic exercise and/or participate in sports. In addition, it remains to be elucidated if the larger volumes and surface areas seen in the current cross-sectional study are due to potential changes in developmental plasticity resulting from aerobic fitness, such as: (1) a larger peak in surface area or volume (e.g., “larger proliferation of synapsis”) during early adolescence; (2) less of an age-related decrease in surface area or volume (e.g., “less pruning”) from adolescence into adulthood, or both. Along these lines, animal studies support the idea that voluntary exercise may increase visual cortex gray matter volumes, as well as increase plasticity in the visual cortex. Sumiyoshi et al. (2014) found that wheel running in rodents from birth to adulthood resulted in prolonging plasticity in the visual cortex, as seen by an extension of the sensitive period of plasticity into adulthood that allowed for a shift in ocular dominance following monocular deprivation (Kalogeraki et al., 2014). While acute effects of exercise do not necessarily translate to long-term chronic effects, a few human adult studies also suggest that, at least acutely, aerobic exercise does in fact change occipital function as seen by functional MRI (Yagi et al., 1999; Li et al., 2014). Thus, longitudinal exercise intervention studies that follow the same individuals over time will be essential to determine if chronic exercise during childhood and adolescence may contribute to previously reported improvements in cognition and brain function through changes in developmental trajectories and prolonged neuroplasticity across development.

The current study also provides preliminary results on how associations between aerobic fitness and brain structure may vary based on an individual’s BDNF genotype. It is important to note that the current study is limited by its small sample size, and only large effect sizes could be detected. As such, the interaction between BDNF genotype and aerobic fitness on bilateral lingual surface area suggests that future research examining the interactions between aerobic fitness and BDNF genotype in larger samples is warranted in order to assess small or medium effects of BDNF genotype that may have been undetectable in the current study. Despite the sample size limitation, these preliminary findings highlight that cortical development is likely a complex and dynamic process influenced by genes, environment, and their interactions (Lenroot et al., 2009). In fact, recent studies have shown that the relative influence of genes verus environment (and their interaction) on cortical development varies across brain region and with age (Lenroot et al., 2009). That is, motor and sensory regions that develop relatively earlier in development (Gogtay et al., 2004) show greater heritability early in childhood, whereas high-order prefrontal and parietal regions show increasing genetic effects later in adolescence (Lenroot et al., 2009). Beyond BDNF, other genes have also been found to influence the relationship between aerobic fitness and brain outcomes. For example, the apolipoprotein e (ApoE) genotype has been shown to moderate the influence of aerobic exercise on brain structure in elderly and aging samples (Honea et al., 2009). More recently, epigenetic mechanisms and micro-RNA have also been found to be responsive to exercise in brain cells in animals (Denham et al., 2014). Together, these findings suggest that an individual’s genes, as well as the expression of those genes, may be especially important factors in understanding how aerobic exercise may affect the brain. While complex, future studies aimed at clarifying the possible interactive effects of aerobic fitness, various genotypes, and gene expression on neurodevelopment will ultimately help us to determine when, how, and to what degree, aerobic exercise may influence the child and adolescent brain.

In summary, we show for the first time that aerobic fitness in late adolescent males relates to frontal, parietal, and occipital cortex structure. In addition, we provide preliminary findings that BDNF genetoype may moderate the relationship between aerobic fitness and cortical structure in adolescents. Further large-scaled longitudinal studies are needed to further elucidate how aerobic fitness may alter neurodevelopmental trajectories and to what degree an individual’s genes may moderate the effect of aerobic exercise on the adolescent brain.

## Author Contributions

MMH and BJN were responsible for study conception, design, and data acquisition, whereas MMH and MFK conducted structural MRI preprocessing and data analyses. All authors (MMH, MFK, BJN) contributed to interpretation of the data, drafting and revision of the intellectual content, have given approval for publication, and are accountable for all aspects of the work.

## Conflict of Interest Statement

The authors declare that the research was conducted in the absence of any commercial or financial relationships that could be construed as a potential conflict of interest.

## Footnotes

^1^http://surfer.nmr.mgh.harvard.edu
